# Leishmaniases' Burden of Disease: Ways Forward for Getting from Speculation to Reality

**DOI:** 10.1371/journal.pntd.0000285

**Published:** 2008-10-29

**Authors:** Richard Reithinger

**Affiliations:** 1 London School of Hygiene and Tropical Medicine, London, United Kingdom; 2 George Washington University School of Medicine and Health Sciences, Washington, D.C., United States of America; Swiss Tropical Institute, Switzerland

In this month's issue of *PLoS Neglected Tropical Diseases*, Bern et al. [1] review the disease burden of the leishmaniases. In their article, the authors review the clinical pathology and eco-epidemiology of the disease as well as provide us with current data on the morbidity, mortality, and socioeconomic impact of the leishmaniases throughout their endemic range. They conclude that “current methods of assessing disease burden fail to take into account the clinical and epidemiological diversity” of the leishmaniases and the “intense medical, social and economic impact” that they might have in highly affected foci.

Bern et al. [1] rightly argue that estimates of the burden of the leishmaniases, both in terms of morbidity and mortality, or in terms of disability adjusted life years (DALYs), are outdated, and/or it is unclear how currently used numbers are derived. Up-to-date figures would assist in garnering funding and political support for the prevention and control of this neglected tropical disease. Clearly, such revised figures could be obtained in a number of ways, including by (i) discussing whether used estimates are actually accurate and/or up-to-date (referring to the commonly used, below-mentioned figures, whether right or wrong); (ii) reviewing whether the methodology to estimate past or current numbers is adequate; or (iii) describing a way forward to collect data allowing for more robust disease burden estimates. In their review, Bern et al. [1] primarily focus on the former, reviewing reports from several countries showing an increase in leishmaniases case numbers. Based on these reports, the authors conclude that the disease burden of the leishmaniases is inaccurate and out-of-date, and the reader is left thinking that it must be higher than currently acknowledged. Additionally, it is highlighted that the approach and formula to estimate the burden of leishmaniases, in terms of DALYs, is flawed: namely, that the values of the used visceral and cutaneous leishmaniasis disability weight and other input parameters are erroneous. Unfortunately, it is unclear from Bern et al.'s arguments [1] what the value of DALY input parameters should be, and as such, their criticism is similar to the criticisms levelled with regards to the burden of disease of every infectious disease of public health importance [2],[3].

Thus, how does one go about in quantifying the burden of the leishmaniases more accurately? The aim of this viewpoint is to elaborate on points (ii) and (iii) above, which with the data reviewed by Bern et al. [1] will provide a platform from where a more accurate estimate of the leishmaniases' burden of disease can be obtained.

## Distribution, Prevalence, Incidence, and Burden of Disease: Current Status or “When Numbers Are Cited Repeatedly, They Invariably Become Hard Fact”

The leishmaniases are believed to be endemic in 88 countries [4]. Broadly speaking, the leishmaniases can be divided into two larger groups of diseases: visceral leishmaniasis (VL) and cutaneous leishmaniasis (CL) [5],[6]. VL is a chronic, systemic disease characterized by fever, (hepato)splenomegaly, lymphadenopathy, pancytopenia, weight loss, weakness, and, if left untreated, death. CL is generally non-fatal and is characterized by a comparatively benign disease that is limited to the skin and that may spontaneously cure. However, as the review by Bern et al. points out [1], because CL represents most of the leishmaniases cases worldwide and because it may progress to severe disease (e.g., leishmaniasis recidivans, mucosal leishmaniasis) resulting in significant social stigma of the affected population, CL's impact on morbidity and quality of life can be considerable.

Whereas 90% of VL cases reportedly occur in Bangladesh, Brazil, Ethiopia, India, Nepal, and Sudan, 90% of CL cases are believed to occur in Afghanistan, Algeria, Brazil, Pakistan, Peru, Saudi Arabia, and Syria. Over the years, there has been significant discrepancy with regards to the data describing the burden of the leishmaniases [7]–[13]. At present, according to the World Health Organization (WHO) figures, every year it is estimated that 1.5–2 million leishmaniases cases and up to 51,000 deaths due to the leishmaniases occur [4],[12],[13]. A total of 350 million people are at risk of infection and disease, and infection prevalence is estimated at 12 million cases. The leishmaniases are, in terms of DALYs, the third most important vector-borne disease, with an estimated 2.4 million DALYs [13]. These figures have been and are used repeatedly by researchers (including the current author), program managers, and policy makers, even though the source of these data is already more than a decade old [9].

## Putting the Spotlight on Disease Burden Input Parameters

Burden of disease is commonly expressed as prevalence or incidence of disease morbidity and mortality, quality adjusted life years (QALYs) or DALYs (see Box 1 for a glossary of terms). DALYs were established in 1992 during the first Global Burden of Diseases (GBD) study to enable health professionals and policy makers to create a scalable measure of disease impact for all health states, which could be extrapolated to country, regional, or global levels to aid in priority setting enabling more (cost)effective implementation of health programs [14].

To answer the question on how to estimate the leishmaniases' burden of disease more accurately, one has to look at the disease burden input parameters, irrespective of whether it is estimated in prevalence or incidence of leishmaniases morbidity and mortality, or DALYs. Moreover, such analysis should be done, whether or not the formula to calculate DALYs is deemed valid or complete [2],[3]. Assuming that some of the generic input parameters (i.e., standard life expectation, age weight, future discount) [2],[3],[14] to estimate the leishmaniases' burden of disease in terms of DALYs are beyond discussion, one is left with four disease-specific input parameters that warrant special attention: age at death, prevalence of disease, duration of disease, and disability weight.

**Box 1. Leishmaniases' Burden of Disease: Definitions**

***Prevalence.*** Actual number of cases of disease present in a population at any particular moment in time.

***Incidence.*** New cases of disease occurring in a specified population in a given time period.

***DALY.*** Disability adjusted life year. A measure of the gap in healthy years of life lived by a population as compared with the normal standard. Essentially, DALYs are a time-based measure that adds together years of life lost due to premature mortality with the equivalent number of years of life lived with disability or illness.

***Disability Weight.*** Measure of the relative valuations of a health state on an interval scale. The disability weight quantifies judgements about overall levels of health associated with different health states, not judgements on the relative lives lived, persons, or of overall well-being, quality of life, or utility. The weights are intended to reflect average global valuations; values are between 0 (i.e., state comparable to ideal health) and 1 (i.e., state comparable to death).

***Discounting.*** Process applied to costs, benefits, and outcomes based on the concept that there is preference for money in health in the present and relative future.

***Duration of Disease.*** Average duration of disease (or disability) in years until remission or death.

### 

#### Age at death and mortality

Compared to other major vector-borne diseases and in the absence of major epidemics (e.g., as the one in Sudan during 1980s that is presumed to have caused up to 100,000 deaths among 280,000 people) [15], mortality due to the leishmaniases has so far been assumed to be limited to VL, with all 51,000 fatalities reported due to VL [13]; fatalities due to CL are very rare and usually due to co-infections or treatment complications [6].

Age at death varies according to the endemic setting, with younger age groups affected in established VL transmission settings and older age groups affected in new VL foci. Thus, the GBD study assumed that 10% and 49% of deaths occurred in age groups <5 and <15 years of age, respectively [14]. Unfortunately, no comprehensive data sets exist that could assess whether this distribution is valid, but it is known that VL incidence is substantially greater in younger age groups if malnutrition is present [16].

A recent study has shown that fatalities due to VL have probably been drastically underestimated. Thus, reviewing clinical records at clinics in Southern Sudan, a study by Collin et al. [17] estimated that 91% of deaths from VL were not reported and, thus, undetected. Also, it has become evident in the past decades that both CL and particularly VL have become an opportunistic infection of HIV/AIDS patients [5],[6], often contributing to their demise, with VL increasing the risk of mortality by more than 3-fold. [18]

#### Prevalence of disease

Although the leishmaniases are endemic in 88 countries, they may not be a notifiable disease in many of them. Also, because of the comparatively benign nature of CL, inaccessibility of health services in rural, endemic areas, and the common non-availability of treatment, severe under-reporting of the leishmaniases is observed (e.g., by a factor of 1∶40 for CL in Guatemala) [19].

Moreover, the leishmaniases, particularly VL, have similar clinical symptoms as other, more prevalent diseases in endemic areas, including malaria and schistosomiasis—to what extent VL is misdiagnosed as, for example, these two diseases is not known. Co-infection with other diseases are possible, as recently shown in Uganda, where malaria was diagnosed in 6.4% of VL cases attending clinical facilities for VL treatment [20].

It is difficult to ascertain whether the estimate of 1.5–2 million annual cases of CL and VL is correct. As mentioned above and highlighted in the review by Bern et al. [1], passive case detection lacks sensitivity and large-scale leishmaniases prevalence surveys are scarce. Of note, however, is that according to the GBD study, for the leishmaniases 66 data sources were included to yield used estimates [14], compared to 282, 117, 89, and 55 for dengue, malaria, lymphatic filariasis, and schistomiasis, respectively. As is highlighted, most of that leishmaniases data appears to be based on an “approximate estimate from current WHO database”, because “original extraction from surveillance data source is not available” [14].

#### Duration of disease

Again, it is unclear what input parameter values were used for duration of disease in current leishmaniases disease burden estimates.

For VL, duration of disease can be up to 2.5 months until diagnosis and treatment [17], depending on infecting parasite species, genetic host factors, and immunosupression (e.g., due to malnutrition or HIV/AIDS). If not treated, VL patients rarely spontaneously cure, with 95% case fatality rates reported [5].

For CL, duration of disease is much more variable, due to the greater number of species causing disease. [6] Thus, whilst clinical disease can spontaneously cure within 2–6 months when due to some species (e.g., *Leishmania major*), it can become chronic if not treated and result in more severe clinical disease (e.g., leishmaniasis recidivans and mucosal leishmaniasis) when due to others (e.g., *L. tropica* and *L. braziliensis*). [6] For most *Leishmania* spp. causing CL, duration of disease is greater than 6 months if not treated.

From current DALY disease burden estimates, it is unclear whether duration of disease includes only active disease (i.e., lesions) or also CL scars—as mentioned below, CL scars can have as big of a social impact as active disease and, hence, duration of disease should be considered “life-long” from time of appearance of CL lesions rather than just being limited to the period of active disease.

#### Disability weight

The disability weight for VL and CL is 0.243 and 0.023, respectively. As Bern et al. point out [1], where these figures come from and how these weights were calculated is somewhat ambiguous, with “major uncertainties” and “sparse documentation” associated with these figures. Whilst many of the infections due to *Leishmania* spp. can be benign, there is now increasing literature on the social impact of the leishmaniases, particularly CL [6]. As in many endemic settings cases do not have access to prompt diagnosis and treatment, CL lesions can be of considerable size and number, and may last for some considerable time (see above), all of which may affect treatment response. Even with successful treatment, a typical CL scar results, which, depending on location (e.g., lesions commonly are on the face due to the exposure to the sandfly vector), *Leishmania* etiology, and type of clinical disease (i.e., localized CL versus leishmaniasis recidivans versus mucosal leishmaniasis), can lead to significant social stigmatization [21]. It would be fair to say that compared to this *emotional* disability, the leishmaniases' impact on *physical* disability is, overall, moderate. For VL, impact of infection and disease can be considerable, with physical disability affected by the characteristic clinical signs (i.e., anemia, hepatosplenomegaly). For CL, physical disability is limited, including at most minor incapacitation of movement or manual labor (e.g., if lesions are located on joints); breathing, swallowing, and talking (e.g., if extensive mucosal leishmaniasis affects mucosae or vocal cords); or urination (e.g., if lesions are located on sexual organs). For both VL and CL, physical disability will also occur if patients undergo the lengthy anti-leishmanial treatment. Depending on the treatment approach and route of administration used, treatment may have considerable (toxic) side effects (e.g., myalgia, gastroenteritis, pancreatitis, hepato and cardiac toxicity, diabetes) [5],[6], affecting a patient's physical condition. Finally, due to clinical disease, duration, or cost of treatment, the leishmaniases may cause considerable *economic* disability, with most treatment approaches exceeding US$200 per patient treated (note, even though treatment in many countries is officially free-of-charge, this often is not the case in practice). Thus, a recent study from the Indian sub-continent suggested that, from a household perspective, each episode of VL was estimated to be associated with US$217 worth of out-of-pocket expenses and loss of income, representing 71% of annual household income [22]. Note, whilst the GBD study specifically states that it does not capture diseases' economic burden (i.e., a different approach would be required to estimate economic loss suffered due to the morbidity or mortality incurred by a disease or condition) [14], it would be comparatively easy to include in the burden calculations direct costs associated with treatment.

In the absence of clear knowledge of how the leishmaniases disability weights were estimated, and taking into account the leishmaniases' impact on emotional, physical, and economic disability, what should the leishmaniases' disability weight be? Without addressing qualitatively and quantitatively the above issues of emotional, physical, and economic disability first, this would be hard to determine. Taking the current disability weight as a benchmark, VL is in the range of disabling leprosy (disability weight: 0.152), malaria episodes (0.191), dengue hemorrhagic fever (0.210), onchocerciasis resulting in low vision (0.260), and trachoma resulting in low vision (0.278) [14]. CL, on the other hand, is in the range of malaria-induced anemia (0.012), hookworm-induced anemia (0.024), onchocerciasis-induced itching (0.068), and lymphatic filariasis characterised by hydroceles (0.073) [14].

## A Way Forward

In light of the above and complementing the recommendations by Bern et al. [1], the following should be the minimum that would be required to obtain an up-to-date estimate of the leishmaniases' burden of disease.

First, a clear understanding should be obtained as to how current estimates of the leishmaniases' burden of disease were obtained, both in terms of case numbers as well as DALYs. Moreover, clarification should be obtained about the nature and origin of input parameters (e.g., whether duration of disease for CL includes scars or only the duration of active CL lesions; specifying the values of each input parameter), and how the disability weight for the leishmaniases was computed. If necessary, disease burden estimates should be amended so as to include the duration of active disease *and* scars for CL, as well as the physical and emotional disability incurred by both VL and CL; if feasible, the economic disability incurred by VL and CL should also be included.

Second, there should be a recommendation, internationally recognized and endorsed, on uniform leishmaniases case definitions and clinical and non-clinical leishmaniases diagnosis algorithms, as well as standardization of approaches to get active and passive case detection estimates in endemic settings. Such harmonization and standardization would ensure that collected data is comparable across endemic countries as well as enable the provision of robust data sources for future burden of disease calculations.

Third, whilst a leishmaniases surveillance system [1] would be laudable, due to cost constraints it is probably not feasible in practice—instead, there should be advocacy to integrate the leishmaniases with other disease surveillance programs (e.g., malaria or Chagas disease), particularly if these have established sentinel surveillance sites.

Fourth, representative surveys (e.g., similar to the Malaria Indicator Surveys developed under Roll Back Malaria's Monitoring and Evaluation Reference Group [23]) should be carried out at regular intervals. Such surveys, together with surveillance data (see above), would allow for more precise estimates of morbidity and mortality of the leishmaniases across endemic regions, prevalence of co-infections, leishmaniases-associated disability, and knowledge of the disease diagnosis, treatment, prevention, and control. A step in this direction is the developed protocol to evaluate neglected tropical disease control programs at the country level [24].

Fifth, sensitivity and specificity analyses should be carried out using a range of input parameters to determine more robust estimates of the disease burden in terms of DALYs, as well as to show how input parameters affect current estimates.

## Conclusion

As with other diseases where the burden of disease was re-assessed (e.g., schistosomiasis, rabies, diarrhoeal diseases) [25]–[27], there is a need to obtain to obtain up-to-date data on the leishmaniases' burden of disease. Only then can a cohesive global leishmaniases prevention and control strategy be formulated, advocacy be done at both fundraising and political levels, and efforts be implemented to significantly impact disease morbidity and mortality, and, hence, burden of disease.
